# Developing a global medicine student pre- and post-travel curriculum

**DOI:** 10.1186/s12909-023-04606-5

**Published:** 2023-10-06

**Authors:** Natasha Mehta, Caroline Fernandes, Christopher Llerena, Stevan Weine, Maarten C. Bosland

**Affiliations:** 1 Department of Internal Medicine, Stanford University, Stanford, CA 94304 USA; 2 Center for Global Health, University of Illinois at Chicago, Chicago, IL 60612 USA; 3 Department of Psychiatry, University of Illinois at Chicago, Chicago, IL 60612 USA; 4 Department of Pathology, University of Illinois at Chicago, Chicago, IL 60612 USA

**Keywords:** Global health, Medical education, Predeparture preparation, Short-term training, Curriculum development

## Abstract

**Background:**

The popularity of short-term global health experiences amongst US medical students has been increasing. However, it remains a challenge for medical schools to comprehensively prepare students to work in an international environment and to contribute in ethically responsible and meaningful ways. Students of the Global Medicine program (GMED) of the UIC College of Medicine Center for Global Health set out to develop a pre-and-post travel curriculum that addresses some of these challenges.

**Methods:**

The students surveyed the literature of 66 published global health curricula and identified aspects of pre-and-post travel training that were found to be under-addressed. They then developed a curriculum in conjunction with GMED faculty that incorporated these identified aspects of pre-and-post travel training.

**Results:**

Five aspects of pre-and-post travel training were identified as being under-addressed in the literature while traveling. These domains include: [1] examining power relations associated with neo-colonization between and within countries; [2] training for bi-directional learning; [3] examining motivations and goals for participating in global health; [4] addressing personal resiliency and psychosocial wellbeing related to students’ travel, and; [5] reflecting on the challenging aspects of the fieldwork experience.

**Conclusions:**

The student-driven curriculum is being integrated into the GMED program through structured didactic sessions, one-on-one mentor meetings and small group discussions. Once students have traveled, the curriculum will be evaluated with the foreign partners they visited.

**Supplementary Information:**

The online version contains supplementary material available at 10.1186/s12909-023-04606-5.

## Background

Over the past few decades, global health has been a growing academic interest among medical trainees in the “Global North” (North America, Europe and other industrialized countries) [1, 2]. Learning about and addressing global health disparities is a passion for many medical students. Medical schools across the world, predominantly in the Global North, have established programs to provide opportunities for global health engagement and research, particularly in the “Global South” (Sub-Saharan Africa, Asia, South America and other developing countries). One in four US medical students has participated in a global health experience at some point in their training [2]. Though experiences can vary in length, purpose, and scope of work, immersion and partnership outside of the classroom and abroad can be an effective tool for many learners to promote reflection, appreciate social determinants, and facilitate professional development [3, 4]. Thus, global health educators are faced with the challenge of preparing students for short-term international electives and ensuring that students have competencies that allow them to contribute in an ethically responsible and meaningful manner [5, 6].

Though the global health field recognizes that pre-departure training should be given to all those working in an international capacity, there is generally no accepted way to provide this education. Historically, pre-departure preparation has focused on logistics such as vaccinations, biomedical training, and preventing infections [7–9], though few have developed guidelines on best practices for interactions between institutions and their trainees [10, 11]. We conducted a review of the literature relevant to pre-departure training and academic global health medicine programs. This demonstrated that while medical students receive training on project-specific knowledge, safety, and ethics, available curricula do not adequately address aspects of power relations and neo-colonization, need for bidirectional learning, personal motivation for global health, resilience and psychosocial wellness, and post-return reflection on challenges during the experience [3]. A new curricular approach is needed to better address these topics of attention in global health travel by medical trainees within pre-and post-travel training. Addressing these gaps in global medical education requires providing skills intended to benefit medical student trainees and the partner organizations with whom they work. We developed additional sessions in the overall global health curriculum for global medicine (GMED) students at the University of Illinois at Chicago College of Medicine (UIC) with the objective of addressing the five identified key gaps in the pre-departure and post-travel training literature.

## Methods

Three students in the GMED program of the Center of Global Health (CGH) at UIC, in conjunction with three GMED faculty, developed a comprehensive pre- and post-departure curriculum for all students in the GMED program prior to traveling for field work and after returning. The PRISMA Checklist was used to guide this literature review. These students conducted a review of the literature and other resources and had discussions with peers and faculty members as a basis for the formulation of a pre- and post-departure curriculum which began being implemented during the 2021–2022 academic year. A literature review was last conducted in December 2022 on PubMed using search terms “pre-departure,” “training,” “global health,” and “students” in all possible combinations [12]. A total of 66 papers were identified with these search terms. With all four search terms, 29 papers were identified; leaving out “students” from the search there were 45 hits; leaving out training there were 30 papers; and leaving out “global health” we obtained 49 hits. Of these 66 papers, only twelve address one or more topics specifically relevant to pre-departure training for global medicine students [3, 4, 7–9, 13–19]. Two of these papers were reviews; the papers addressed either specific topics or an overall curriculum [3, 7]. In addition, global health websites from some large academic institutions known to have existing global health trainee programs (Vanderbilt University, Johns Hopkins University, and the University of Chicago) and organizations (American Medical Student Association, Unite for Sight) in the United States were searched for information on published pre-departure curricula.

The students collaborated with a parallel student-faculty group intended on bringing a focus to decolonization and anti-racism to the GMED curriculum which is comprised of clinical and research faculty associated with the Center for Global Health and School of Medicine with several years of experience working with partners in the “Global South”. The students regularly met with GMED faculty mentors to discuss literature findings and best practices identified in programs at other institutions. The student team devised topics, learning objectives, and structured activities broken into two pre-departure sessions and one post-return session of two hours each.

## Results and discussion

Our review identified five key gaps in the pre-departure and post-travel training literature: [1] examining power relations associated with neo-colonization between and within countries; [2] training for bi-directional learning; [3] examining motivations and goals for participating in global health; [4] addressing personal resiliency and psychosocial wellbeing related to students’ travel, and; [5] reflecting on the challenging aspects of the fieldwork experience. These five pre- and post-departure issues in global health training of medical students are discussed in the following sections.

**Fig. 1:**
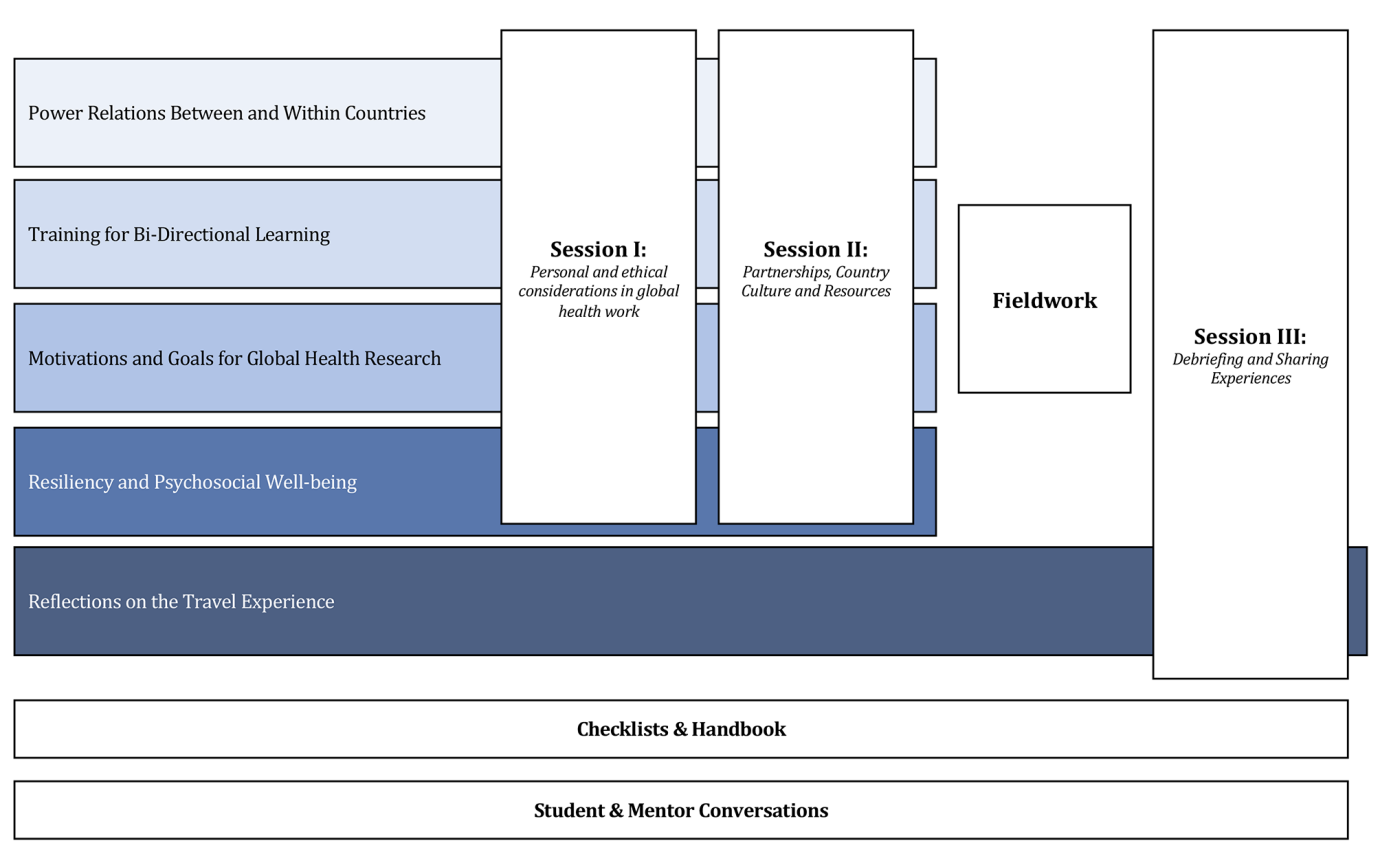
Curricular Map and Session Plan

Drawing from examples in the literature and other programs, the structure of components of each session ranged from didactic talks, discussion groups, case studies, and individual reflective assignments [7]. The curricular plan and road map to implement it were presented to the GMED education team faculty and core CGH faculty for feedback in a series of meetings (Fig. 1). The proposed curriculum is presently being implemented in the GMED curriculum. Additionally, as a resource for this curriculum, the students developed a handbook that included important documentation about travel policies, links to country-specific information, and international best practices on safety, as well as a checklist of topics mentees should discuss with their mentor, a core faculty member, prior to traveling.

### Power relations between and within countries

Partnerships between the “Global North” and “Global South” must work to promote equity and to combat racism and exploitation rooted in colonialism and imperial motives. Increasingly, students from high-income countries (HICs) are urged to learn about the historic context of global health when being trained to work with populations in low- and middle-income countries (LMICs) [4, 5]. However, understanding the impact of preexisting North-South and HIC-LMIC relationships and the development of critical thinking skills for examining power relations are often not included in training global health students [5].

The asymmetric power structures created by colonial rule continue to remain pervasive in the present field of global health and have been termed “neo-colonialism.” In response, a broader call has evolved for programs to address or “decolonize” their approach to scholarly work [5, 20, 21]. Within the context of global health work, this concept is important to acknowledge as it directly impacts funding, research agendas, development of local infrastructure and much more [8]. In order for students to act in line with this goal, it is essential for them to develop a keen understanding of decolonization by examining power dynamics and development narratives specific to the contexts in which they are working.

In our curriculum objectives, we address competencies related to understanding the political, social, and cultural history of the country students are working in as well as defining and reflecting on the scope of practice while engaging in global health work. In the second session, students are asked to research the political and cultural history, language, disease burden and governing health agencies of their country or region of focus with the assistance of the mentor of their research project. Recognizing that fully learning one county’s in-depth history is not possible for students to achieve on their own, mentors are tasked with assisting trainees to focus on topics most relevant to their work. This occurs over a series of individual conversations built into the GMED mentorship program. Students are also asked to outline potential ethical issues in their research proposal during mentor meetings to allow for feedback and adjustment prior to departure [13]. Additionally, students are brought together in a group setting to share information with the other GMED students about the culture in which they are working. This activity builds a community of students committed to critical engagement with the practice of global health. These discussions provide a space in which students can learn about several global communities and discuss challenges they may face in navigating the dynamics within the international team. By the end of these exercises students will be able to describe how neo-colonialism impacts their research and identify concrete means through which their project will strive to uphold decolonialist values.

### Training for bi-directional learning

The historical issue of power dynamics between the Global North and South or other patterns of domination or oppression oftentimes results in trainees from the former unilaterally benefitting from their global health experience with little exchange of knowledge with local partners. Existing curricula do not adequately address the essential goal of bi-directional learning and information exchange between partners [4]. Additionally, few programs have reported host community perspectives on the benefits of international student electives [16]. Thus, it is critical to make partnership and goal alignment core components of any pre-departure curriculum.

Our curriculum’s learning objectives in this domain address recognizing and understanding the scope of practice for medical trainees working in an international setting in addition to developing skills to promote bidirectional knowledge gain. Designed as a session with faculty and research partners from various LMIC sites and a subsequent small-group discussion amongst students, the learning objectives include building trust with LMIC partners and communicating joint lessons learned, acknowledging limitations in resources, skills, knowledge and abilities, and applying leadership practices that support team effectiveness and building trust between partners.

Organized as a panel, we ask LMIC partners to share their experiences with GMED students, paying particular attention to the role of global health trainees and identifying areas for improvement of relationships between HIC and LMIC institutions. Global health experiences often focus on US trainees that are traveling to LMICs to learn. Therefore, we also ask LMIC partners to identify and discuss opportunities for their constituents to learn from our students while recognizing the limitations of this in the LMIC context. LMIC research partners are asked to guide students on creating and setting goals together, encouraging representation and input from all stakeholders. Furthermore, LMIC partners are asked to share their perspectives on the scope of practice medical trainees should have while working within their culture. Students also discuss their specific clinical role and bidirectional learning opportunities with their individual mentors, since the details of training, the role of the medical student trainee, and ethical responsibilities will differ between sites. As part of this discussion, feedback from their LMIC partner will be sought via Zoom, phone, or email. Additionally, students are asked to reflect on the power dynamics that may be at play during the panel discussion with the LMIC partners. These efforts to ensure the value of the project to host country avoid the well documented phenomenon of “medical volunteerism” [16]. Through these activities students and LMIC partners will build a reciprocal working relationship that can further the personal and academic agendas of all persons involved.

### Motivations and goals for global health research

All students have their own journey that led them to pursue global health as a career path during medical school. These interests and personal motivations impact which mentors and projects students seek. Self-reflection is an important but often overlooked aspect in the process of designing and implementing a global health project. In addition to providing students with information about the culture, history, and partnerships within which medical students will work, it is critical to focus on the trainees themselves [3–7]. To facilitate personal goal setting for these students, they must first examine their own motivations and reasons for their interest in global health and internships in LMICs, an exercise often missing from global health curricula.

Learning objectives aimed at this domain include identifying strategies to align personal goals with program goals, in addition to defining personal motivations and interest for a career in global health beyond potentially reductionist ideas of charity, aid, and altruism, recognizing the difference between motivation and good intentions [20]. The curriculum does not specifically address these issues in the two pre-departure session as self-reflection is incorporated in the student’s initial capstone project statement, their presentations to the GMED faculty during first and fourth years, and a post-return reflection session, in addition to ongoing conversations with mentors. Collectively, these statements and discussions provide core global medicine faculty a chance to assess students’ motivations for global health research and reinforce the idea that a commitment to global health involves a continuous process of learning, unlearning, and relearning [20]. These activities also challenge students to decenter themselves from motivations to pursue global health work and instead adopt a framework of global solidarity [20]. In this way, solidarity goes beyond aid or charity and instead assumes a commitment to eliminate oppressive systems and lessen suffering [20].

By the end of these activities, students prepare a statement regarding their motivations for their research project interest to be presented during their capstone proposal, final presentations and reflection papers in their first and fourth years, respectively.

### Resiliency and wellness

In recent years, student mental health and wellness has become a topic of interest to educators and is now a particular focus in medical school training [22]. While a larger supportive culture emphasizing student well-being had been established within the UIC GMED program, the existing curriculum lacked the space or tools for students to build resiliency in the setting of their global health work. International travel can be accompanied by unfamiliarity with new foreign cultures and may be a time when students’ mental health and overall wellness are challenged [3, 9]. Acculturation, the process of adjusting to a different environment, can raise feelings of frustration, anxiety, irritability, tiredness, and homesickness [13]. Besides addressing acculturation, developing psychological resiliency, the ability to cope with or protect oneself from stressors, is important in preparing for challenging high-demand work associated with research abroad [23]. Few programs address resiliency prior to travel, or reflection after the global health experience [24]. Additionally, the phenomenon of clinical acculturation, adjusting to differences in clinical medical practice, poses a similar set of challenges, particularly for those directly involved with clinical care or concurrently training in a different clinical setting. This is rarely addressed in pre-departure training but is important in recalibrating how clinical care is provided in different cultural contexts. Consideration of clinical acculturation is incorporated into the pre-departure handbook and the curriculum. These materials cover the scope of practice of the trainee abroad and how to process and cope with differences in medical care standards [25].

The curriculum focuses on the learning objective of defining and normalizing emotional and moral distress during global health work and developing a resiliency strategy for staying well while working outside of the US [26]. One key aspect is creating a space for students to reflect on their personal challenges with developing resiliency. We formally introduce a discussion around resiliency and wellness during the first pre-departure session. During this exercise, faculty members lead a discussion around cultivating emotional resilience prior to travel and creating strategies to adapt effectively to foreign cultures. These strategies include anticipating and self-identifying signs of mental distress and creating a multimodal psychosocial wellness plan that may include journaling, exercise, and reaching out for support. Additionally, this session aims to identify factors that can contribute to or challenge individual well-being while traveling. This includes aspects of health, personal safety, and communication with LMIC team members and mentors. Lastly, students are required to discuss with their mentors their personal mental health plan in addition to the pre-departure sessions. Furthermore, during the second pre-departure session during the panel with local, on-site partners, trainees and mentors engage in conversations regarding the scope of practice and differences they may experience in the clinical setting. Students are thus primed to think about how they might address differences in clinical practice styles prior to arriving on site. These conversations continue through the one-on-one faculty and local mentor meetings.

### Reflection on the travel experience upon return

Arguably one of the most important aspects of international research is the reflective process upon return. We included formal components in the curriculum to specifically facilitate and encourage reflection on the travel experience related to the four topics detailed above and the actual global health experience.

Soon after their return, each student has a formal debriefing with their mentor to reflect on their specific personal experiences with feedback from their LMIC partner. Once all students have returned from their travel, typically during the summer between the M1 and M2 years, a post-departure session is held where students collectively reflect on and share their experiences, including successes, failures, and ethical issues, focusing on the pre-departure issues with the aim to exchange their experiences. This exercise encourages students to deepen their perspective of individual challenges faced as well as learn from other students’ unique experiences. Structured as a narrative medicine session, students are prompted to write and reflect on their experiences including areas of success as well as clinical and personal challenges prior to this final session and are asked to share their narratives in the session with their peers. The objectives of the session are to acknowledge personal limitations in knowledge and abilities, reflect on building partnerships, and communicate joint lessons learned with partners, peers and the program. The session is facilitated by a faculty member with experience in leading narrative medicine sessions.

Additionally, faculty leadership lead a structured debrief on physical and psychosocial safety for the students within the post-return session. Lessons learned can both enrich the individual student’s understanding of global health, working abroad, and provide information to improve the curriculum and foster long-term partnerships with host communities. Actively involving faculty in these “lessons learned” provides longitudinal institutional knowledge to the GMED students, program faculty and other medical students engaging in global health work. An additional major goal of this session is to identify concrete ways for the GMED program to improve future visits for students and local communities. Upon the conclusion of these activities, students prepare a reflective report of their experience to be included in their final capstone presentation.

## Conclusions

Short-term global health experiences have the potential to establish productive collaborations, promote innovative research and enhance the career development of medical students. However, new activities are needed to prepare these students for short-term experiences within the larger trajectory of their global health work. The curriculum gives students the opportunity to develop their long-term global health motivations and goals and share them with mentors, international partners, and peers as they prepare their projects, as well as to reflect on the challenges of their experiences upon return. This curriculum aims to emphasize decolonialism, bi-directional learning, acculturation, building trust with LMIC partners, and psychosocial wellbeing and developing resiliency for students to have the necessary tools when facing challenges in their work. Furthermore, new challenges, such as SARS-CoV-2 (COVID-19), may emerge that require novel adaptations to existing curricula [27, 28]. Although these sessions are aimed toward preparation for field work, challenges like COVID-19 may necessitate training students to collaborate with partners remotely while maintaining equitable practices [27]. As this curriculum continues to be implemented within the larger UIC GMED framework, it will necessarily undergo evaluation and further changes given the overall GMED curriculum’s nature of continuing development with its objective to better equip students to become leaders within the dynamic and continuously evolving field of Global Health. In particular, an evaluation of the effectiveness of this student-driven curriculum will be conducted with the LMIC partners who hosted and mentored students while abroad. We hope that the UIC GMED curriculum will serve as an example to other institutions and encourage further innovation to establish best practices in this space.

### Electronic supplementary material

Below is the link to the electronic supplementary material.

Supplementary Material 1

## Data Availability

All data is available upon request. Please contact Natasha Mehta (namehta@stanford.edu) for data.
